# Cerebrovascular and ventilatory responses to acute normobaric hypoxia in girls and women

**DOI:** 10.14814/phy2.13372

**Published:** 2017-08-03

**Authors:** Laura E. Morris, Daniela Flück, Philip N. Ainslie, Ali M. McManus

**Affiliations:** ^1^ Centre for Heart Lung and Vascular Health School of Health and Exercise Sciences University of British Columbia Kelowna Canada

**Keywords:** Cerebral perfusion, children, hypoxia, respiratory drive, ventilation

## Abstract

Physiological responses to hypoxia in children are incompletely understood. We aimed to characterize cerebrovascular and ventilatory responses to normobaric hypoxia in girls and women. Ten healthy girls (9.9 ± 1.7 years; mean ± SD; Tanner stage 1 and 2) and their mothers (43.9 ± 3.5 years) participated. Internal carotid (ICA) and vertebral artery (VA) velocity, diameter and flow (Duplex ultrasound) was recorded pre‐ and post‐1 h of hypoxic exposure (FIO_2 _= 0.126;~4000 m) in a normobaric chamber. Ventilation (${\dot{V}}_{E}$) and respiratory drive (*V*_T_/*T*_I_) expressed as delta change from baseline (∆%), and end‐tidal carbon‐dioxide (P_ET_CO_2_) were collected at baseline (BL) and 5, 30 and 60 min of hypoxia (5/30/60 HYP). Heart rate (HR) and oxygen saturation (SpO_2_) were also collected at these time‐points. SpO_2_ declined similarly in girls (BL‐97%; 60HYP‐80%, *P *<* *0.05) and women (BL‐97%; 60HYP‐83%, *P *<* *0.05). Global cerebral blood flow (gCBF) increased in both girls (BL‐687; 60HYP‐912 mL·min^−1^, *P *<* *0.05) and women (BL‐472; 60HYP‐651 mL·min^−1^, *P *<* *0.01), though the ratio of ICA:VA (%) contribution to gCBF differed significantly (girls, 75:25%; women, 61:39%). The relative increase in ${\dot{V}}_{E}$ peaked at 30HYP in both girls (27%, *P *<* *0.05) and women (19%, *P *<* *0.05), as did ∆%*V*_T_/*T*_I_ (girls, 41%; women, 27%, *P*'s < 0.05). Tidal volume (*V*_T_) increased in both girls and women at 5HYP, remaining elevated above baseline in girls at 30 and 60 HYP, but declined back toward baseline in women. Girls elicit similar increases in gCBF and ventilatory parameters in response to acute hypoxia as women, though the pattern and contributions mediating these responses appear developmentally divergent.

## Introduction

In adults, exposure to acute hypoxia results in rapid declines in the partial pressure of arterial oxygen (PaO_2_), a compensatory increase in ventilation (${\dot{V}}_{E}$) and, as a consequence, declines in the partial pressure of arterial carbon dioxide (PaCO_2_) (Weil et al. 1970). These changes are closely coupled with the regulation of cerebral blood flow (CBF), such that when PaO_2_ falls below ~50 mmHg, at least in adults, cerebral vasodilation occurs and perfusion is augmented (Willie et al. 2012; Lewis et al. 2014; Hoiland and Ainslie 2016). This vasodilatation occurs throughout the cerebrovascular tree, from the large extracranial and intracranial conduit arteries (e.g., the internal carotid (Willie et al. 2012) and middle cerebral arteries (Wilson et al. 2011; Imray et al. 2014)), to the arterioles in the pial mater (Wolff et al. 1930). Evidence of the ventilatory and cerebrovascular responses to hypoxia in children is sparse (Kohler et al. 2008; Gavlak et al. 2013), with much of our understanding extrapolated from adult data. This is problematic because of documented developmental differences in the ventilatory (Gratas‐Delamarche et al. 1993) and cerebrovascular responses of the child (Schöning and Hartig 1996).

Evidence that the ventilatory response to hypoxia is dependent on age is mixed. Using an isocapnic progressive hypoxic technique, no differences in the hypoxic ventilatory response was noted from 7 to 18 years of age (Honda et al. 1986). In contrast, using the same isocapnic rebreathe technique, Marcus et al. (1994) reported a significantly increased hypoxic ventilatory response in children compared to adults; however, the relationship with age was weak (*r* = 0.34). Following 1 day at 3450 m, an increased respiratory drive (*V*_T_/*T*_I_; *V*_T_, tidal volume; *T*_I_, inspiratory timing) and ${\dot{V}}_{E}$ was found in both children and adults, suggesting that ventilatory responses to hypoxia are independent of age (Kohler et al. 2008). Further clarification is needed to better understand how age related differences (present or not) may affect ventilatory responses to an acute bout of hypoxia.

Cerebral perfusion shows distinct developmental patterns, peaking between the ages of 5–10 years, with values 30–50% greater than in adults, and a higher cerebral blood flow in girls compared to boys (Leung et al. 2016). This greater perfusion in children is reflected in a reduced cerebrovascular reserve, as indicated by an attenuated cerebrovascular vasodilation to hypercapnia (Leung et al. 2016). There is very limited data on the cerebrovascular response to hypoxia in the child, with one study demonstrating an increase in perfusion in the anterior cerebral circulation (indexed by increased middle and anterior cerebral artery blood velocity), but not in the posterior  cerebral circulation (indexed by the basilar and posterior cerebral arteries) in children aged 6–13 years who completed a 5‐day ascent to 3500 m (Gavlak et al. 2013). A limitation of this approach is the assumption that vessel diameter is unchanged; however, since hypoxia may lead to dilation of the cerebral arteries (Wilson et al. 2011) this would result in an underestimation of CBF (Ainslie and Hoiland 2014; Hoiland and Ainslie 2016). In adults, increases in global cerebral perfusion in response to normobaric isocapnic hypoxia from about 80% oxygen saturation (SpO_2_), were a consequence of a greater increase in the posterior circulation (via the VA, vertebral artery) compared to anterior flow (via the ICA, internal carotid artery) (Willie et al. 2012; Hoiland et al. 2017). Regional CBF changes in response to acute hypoxia in the healthy child is unknown.

The purpose of this investigation therefore was to determine the ventilatory (${\dot{V}}_{E}$, *V*_T_/*T*_I_), respiratory gas exchange and cerebrovascular (extracranial blood flow and vasodilation; ICA and VA) responses to acute normobaric hypoxia in girls and women, and explore relationships between changes in CBF, SpO_2_, and end‐tidal carbon dioxide (P_ET_CO_2_). Given the documented sex differences in CBF (Tontisirin et al. 2007) and potential hereditary influences on the cardiorespiratory response to hypoxia (Kriemler et al. 2015) we chose to limit our comparison to girls and their biological mothers. We hypothesized that following 1 h of normobaric hypoxia (1) the magnitude of change in ${\dot{V}}_{E}$ and *V*_T_/*T*_I_ would be similar between girls and women, (2) elevations of CBF in girls would be mediated via greater elevations in flow in the ICA versus VA, compared to greater elevations in flow in the VA in women and (3) that increases in CBF would be correlated to declines in SpO_2_ and P_ET_CO_2_.

## Methods

### Participants

Ten healthy pre‐ and early‐pubertal girls (9.9 ± 1.7 years) and their biological mothers (43.9 ± 3.5 years) were recruited. All participants were born, raised, and resided at low altitude with no history of cardiorespiratory, circulatory, or metabolic disease. All girls were classified as Tanner stage 1 or 2 by parental assessment of Tanner stage (Rasmussen et al. 2015). Informed consent was obtained from parents, and written assent obtained from children. Ethical approval was granted by the Clinical Research Ethics Board (H16‐00855).

### Procedures

Girl–mother pairs attended the laboratory once for 2.5 h, located in Kelowna (344 m). The girls and their mothers were tested together to help reduce any anxiety the child may have felt. Participants were asked to refrain from vigorous exercise and caffeine 12 h prior to arrival, and visit at least 2 h postprandial. After familiarization with the experimental procedures, initial anthropometric measurements were taken, followed by 5 min of rest, after which baseline normoxic SpO_2_, heart rate (HR), ${\dot{V}}_{E}$, *V*_T_/*T*_I_ and P_ET_CO_2_ were assessed. Diameter and flow of the ICA and VA were recorded, using Duplex ultrasound. Participants then entered an air tight normobaric hypoxic chamber for 1 h and all measures were repeated following 5 (5 HYP), 30 (30 HYP) and 60 (60 HYP) minutes of hypoxic exposure, with the exception of ICA and VA diameter and flow, which were assessed after 60 min only. Rating of acute mountain sickness (AMS) and cerebral symptoms were completed after 1 h of hypoxic exposure before leaving the chamber. The fraction of inspired oxygen within the chamber was continuously sampled using a gas analyzer (ML206 Gas Analyser, ADInstruments, Colorado) and the chamber was maintained at 12.58 ± 0.1% to simulate ~4000 m altitude.

### Primary measures

#### Respiratory function

Ventilation (breathing frequency [*f*_R_] and *V*_T_), *V*_T_/*T*_I_ and P_ET_CO_2_ were collected continuously for 5 min, by sampling breath‐by‐breath volumes and gas concentrations at the mouth using an online metabolic gas‐analysis system (Oxycon Pro, Care Fusion, Hoechberg, Germany). The children and adults wore a mouthpiece and nose clip and flow volumes were measured with a low dead space (40 mL) turbine. The practicality of using the equation ‘*V*_T_/*T*_I_’ to measure the drive to breathe has been advocated in children, and is directly related to mouth occlusion pressure – a more direct, but invasive measure of respiratory drive (Gaultier et al. 1981). Prior to measurement at normoxic baseline or hypoxia, the digital turbine volume sensor was calibrated with a 3 L syringe and the gas analyzer was calibrated with a known concentration of gas. Data were converted from breath by breath to second‐by‐second over the 5‐min collection period and ventilatory volumes were expressed as a ratio standard with body mass.

#### Extracranial blood flow

Volumetric blood flow of the right ICA and VA was measured, using a 10 MHz multi‐frequency linear array probe and a high‐resolution ultrasound machine (Terason 3000, Teratech, Burlington, MA), while participants lay supine. B‐mode imaging and pulse‐wave velocity was optimized to obtain arterial diameter and blood flow velocity, respectively. All scans were recorded over at least 10 consecutive cardiac cycles, using custom‐designed edge‐detection and wall‐tracking software to determine diameter and flow, as described in depth elsewhere (Thomas et al. 2015). Global cerebral blood flow (gCBF) was calculated using the following formula:

gCBF=2(ICA flow+VA flow)

### Secondary measures

HR was assessed using telemetry (Polar T31, Polar Electro OY) and SpO_2_ by pulse oximetry (MD300K1 Pulse Oximeter, VacuMed, California). The Lake Louise Sickness Score (LLSS; Roach et al. 1993) was used to assess altitude sickness symptoms. The category ‘difficulty sleeping’ was removed since participants did not sleep. A score ranging from 3 to 5 was considered mild AMS and any value exceeding this was considered severe. Participants also rated any cerebral headache they experienced on a scale from 0 to 100; 0 being no headache at all and 100 being the worst headache imaginable on the cerebral‐specific section of the environmental symptoms questionnaire (ESQ‐CS; Sampson et al. 1983).

### Data and statistical analysis

Descriptive data were expressed as means and SD. Ventilatory, respiratory, HR, and SpO_2_ responses to normobaric hypoxia were assessed, using time (baseline, 5, 30 and 60 min exposure) by age repeated measures analyses of variance (RM ANOVA). Where baseline values differed by age percentage change from baseline was calculated and used in the analyses. Extracranial blood flow and diameter were also examined using time (baseline and 60 min) by age (girls, women) RM ANOVA; however, when percentage change from baseline was calculated a one‐way ANOVA was used to compare the hypoxic response. Simple effects, using *t*‐tests were used to deconstruct main effects and interactions from the RM ANOVA where necessary. Statistical significance was set a priori at *P *≤* *0.05. All statistical analysis was performed using SPSS (Statistical Package for Social Sciences).

## Results

Participant characteristics are presented in Table 1. Eight of the ten girls were classified as Tanner stage 1 and two were Tanner stage 2. As expected, height and weight were greater in the women. Of the ten girl–mother pairs, two children had incomplete ventilatory data; and, in one adult, ICA measures were not adequately obtained following 60 HYP.

**Table 1:** Descriptive characteristics

	Girls (*n *=* *10)	Women (*n *=* *10)	Child‐adult difference
Age (y)	9.9 (1.7)	43.9 (3.5)	*P *<* *0.05
Height (cm)	141.2 (11.3)	167.8 (6.3)	*P *<* *0.05
Weight (kg)	34.8 (6.2)	62.4 (11.7)	*P *<* *0.05

Data are mean (±SD).

**Figure 1:**
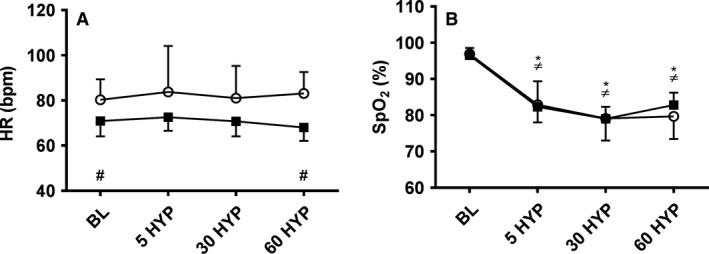
Heart rate (panel A) and oxygen saturation (panel B) at baseline and following 5 (5 HYP), 30 (30 HYP) and 60 (60 HYP) minutes of hypoxia in girls (white circles) and women (black squares); *within subject change from baseline in girls, *P *<* *0.05; ≠within subject change from baseline in women, *P *<* *0.05; #child‐adult difference, *P *<* *0.05.

No significant change in HR was observed in either group (see Fig. 1, panel A). SpO_2_ declined with increasing hypoxia (*F*(3,54) = 74.049, *P *<* *0.01) similarly in girls and women (see Fig. 1, panel B).

There was no difference in LLSS reporting's for girls (3 ± 2) or women (2 ± 2). Three girls presented with mild and one with severe AMS and four women presented with mild AMS. The ESQ‐CS scores were also similar for girls (19 ± 27) and women (18 ± 24).

### Ventilation

**Figure 2:**
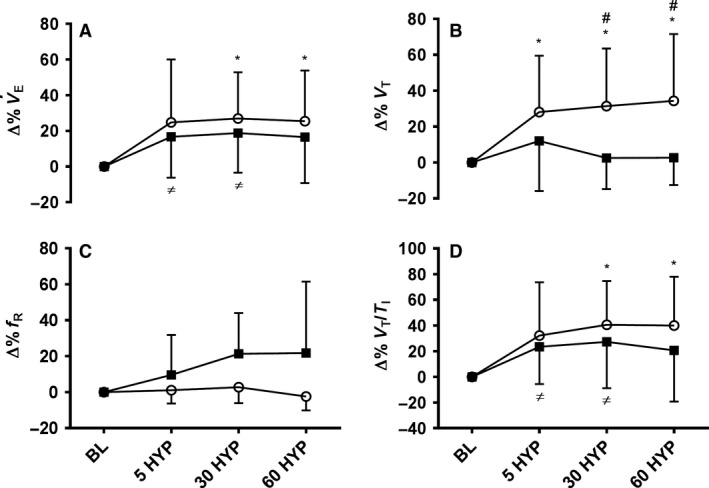
Relative change from baseline in ventilation (Δ%; panel A), tidal volume (Δ%; panel B), breathing frequency (Δ%; panel C) and respiratory drive (Δ%; panel D) following 5 (5 HYP), 30 (30 HYP) and 60 (60 HYP) minutes of hypoxia in girls (white circles) and women (black squares); *within subject change from baseline in girls, *P *<* *0.05; ≠ within subject change from baseline in women, *P *<* *0.05; # child‐adult difference, *P *<* *0.05.

${\dot{V}}_{E}$ (Δ%) increased during hypoxia (*F*(1.897,30.345) = 8.557, *P *<* *0.01), in a similar manner in girls and women (see Fig. 2, panel A). Initial exposure to 5 HYP elicited increases of 25% in girls, although not significantly greater than baseline, whereas at 30 HYP and 60 HYP ${\dot{V}}_{E}$ was significantly greater than baseline. In women ${\dot{V}}_{E}$ was elevated 17% above baseline at 5 HYP (*P *<* *0.05) which remained elevated at 30 HYP (*P *<* *0.05) before returning toward baseline at 60 HYP. ${\dot{V}}_{E}$ normalized to body mass is reported in Table 2.

**Table 2:** Ventilatory responses at baseline (BL), and following 5 (5 HYP), 30 (30 HYP) and 60 (60 HYP) minutes of hypoxic exposure in girls and women

		BL	5 HYP	30 HYP	60 HYP
${\dot{V}}_{E}$ (mL·kg·min^−1^)	Girls	241.8 (62.6)	295.0 (80.3)	302.6 (78.0)^a^	295.3 (65.5)^a^
	Women	119.6 (20.7)^b^	136.7 (19.9)^a^^,^ b	139.3 (20.0)^a^^,^ b	135.7 (14.3)^a^^,^ b
P_ET_CO_2_ (mmHg)	Girls	34.7 (2.9)	30.1 (3.9)^a^	28.1 (3.1)^a^	26.6 (2.1)^a^
	Women	36.3 (2.7)	32.7 (3.0)^a^	30.5 (1.7)^a^	28.3 (1.9)^a^
$\dot{V}O_{2}$ (mL·min^−1^)	Girls	255.0 (52.1)	304.5 (69.6)	355.2 (72.6)^a^	333.9 (70.4)^a^
	Women	275.3 (69.2)	276.0 (65.6)	301.4 (58.0)	298.7 (55.6)
$\dot{V}{\text{CO}}_{2}$ (mL.min^−1^)	Girls	248.8 (65.2)	267.8 (64.9)	253.6 (50.9)	238.5 (53.7)
	Women	234.0 (67.6)	247.0 (57.2)	223.8 (41.0)	202.1 (26.4)
${\dot{V}}_{E}$/$\dot{V}O_{2}$	Girls	31.8 (4.7)	32.3 (3.7)	30.1 (2.8)	29.8 (2.8)
	Women	24.7 (8.6)	28.3 (10.3)^a^	26.3 (9.3)^a^	26.2 (9.3)
${\dot{V}}_{E}$/$\dot{V}{\text{CO}}_{2}$	Girls	32.7 (2.8)	37.0 (5.0)^a^	39.8 (4.5)^a^	41.9 (4.4)^a^
	Women	29.0 (10.4)	31.3 (11.4)^a^	35.2 (12.5)^a^	37.9 (13.6)^a^

Data are mean (±SD).

a Within subject change from baseline, *P *<* *0.05

b Child‐adult difference, *P *<* *0.05. Girls, *n *=* *8; women *n *=* *10.

*V*_T_ (Δ%) increased during hypoxia (*F*(2.026, 32.421) = 6.026, *P *<* *0.01), but in an age divergent manner (*F*(2.026, 32.421) = 3.616, *P *<* *0.05; see Fig. 2, panel B). With initial hypoxia (5 HYP), no difference in *V*_T_ (Δ%) was apparent between girls and women; however, with increasing hypoxic exposure *V*_T_ (Δ%) remained elevated above baseline in girls, and higher than women, whereas *V*_T_ declined back toward baseline in women (*P*'s < 0.05). The pattern of change in f_R_ (Δ%) with hypoxia is shown in Figure 2, panel C. The response was highly variable and as such neither the main effect for hypoxia (*P *=* *0.10), nor the interaction with age were significant (*P *=* *0.097).

### Drive to breathe

*V*_T_/*T*_I_ (Δ%) increased during hypoxic exposure (*F*(1.734,27.740) = 10.134, *P *<* *0.01) similarly for girls and women. In the girls, *V*_T_/*T*_I_ increased by 32% (*P *>* *0.05) from baseline to 5 HYP, becoming significant at 30 HYP (41%, *P *<* *0.05) and 60 HYP (40%, *P *<* *0.05). In women, *V*_T_/*T*_I_ increased by 23% at 5 HYP (*P *<* *0.05), remained elevated at 30 HYP, but was not significantly greater than baseline at 60 HYP (Fig. 2, panel D).

### Respiratory gas exchange

Declines in P_ET_CO_2_ with hypoxic exposure (*F*(1.692,27.075) = 86.946, *P *<* *0.001) were apparent in both girls and women at all time‐points (*P*'s < 0.05; see Table 2).

A main effect for hypoxia was apparent for $\dot{V}O_{2}$ (*F*(3,48) = 8.196, *P *<* *0.001), but there was no main effect for age (*P *>* *0.05) or an interaction (*P *=* *0.08; see Table 2). There was no difference between baseline $\dot{V}O_{2}$ and initial hypoxia (5 HYP) in girls, with $\dot{V}O_{2}$ rising at 30 HYP (*P *<* *0.05) and remaining elevated above baseline at 60 HYP (*P *<* *0.05). In the women, ($\dot{V}O_{2}$ was similar to baseline at 5 HYP, but increased in comparison to 5 HYP by 30 HYP (*P *<* *0.05). Likewise, a main effect for hypoxia was apparent for $\dot{V}{\text{CO}}_{2}$ (*F*(1.909,30.551) = 3.484, *P *<* *0.05), with no main effect for age or interaction (*P*'s > 0.05; see Table 2). Mean $\dot{V}{\text{CO}}_{2}$ was lower in the girls from 5 to 60 HYP (268 mL·min^−1^ vs. 238 mL·min^−1^, *P *=* *0.056), whereas in the women declines were noted from 5 to 30 HYP (247 mL·min^−1^ vs. 223 mL·min^−1^ *P *<* *0.05), and from 30 to 60 HYP (223 mL·min^−1^ vs. 202 mL·min^−1^ *P *<* *0.05).

The ventilatory equivalent for $\dot{V}O_{2}$ also altered with hypoxia (F(2.026,32.418) = 5.849, *P *<* *0.01) and in an age‐specific manner (*F* (2.026,32.418) = 3.310, *P *<* *0.05; see Table 2). In girls, ${\dot{V}}_{E}$/$\dot{V}O_{2}$ was similar to baseline at 5 HYP, 30 HYP, and 60 HYP, although there was a significant fall in ${\dot{V}}_{E}$/$\dot{V}O_{2}$ between 5 HYP and 30 HYP (*P *<* *0.05). In contrast, ${\dot{V}}_{E}$/$\dot{V}O_{2}$ rose in women from baseline to 5 HYP (*P *<* *0.05), remaining elevated above baseline at 30 HYP (*P *<* *0.05). The ventilatory equivalent for $\dot{V}{\text{CO}}_{2}$ was also altered with hypoxia (*F*(2.127,34.037) = 5.849, *P *<* *0.01; see Table 2), but in a similar manner in girls and women. ${\dot{V}}_{E}$/$\dot{V}{\text{CO}}_{2}$ was elevated above baseline at all time‐points in both the girls and women (*P*'s < 0.01).

### Cerebral blood flow

**Figure 3:**
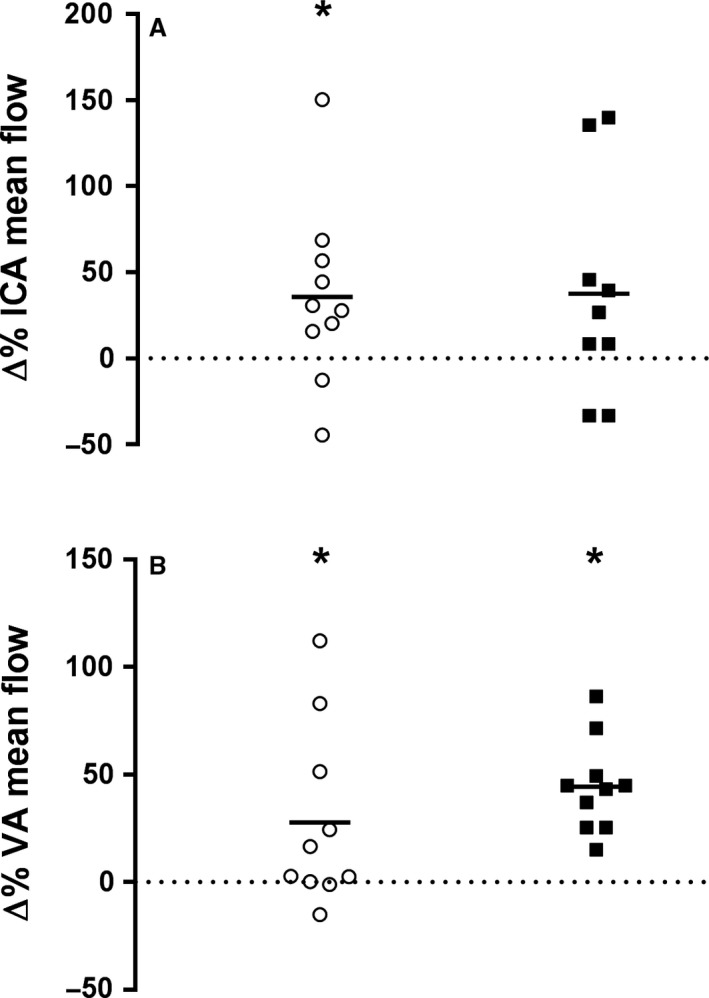
Relative change from baseline to 60 min of hypoxia in internal carotid artery blood flow (Δ%; panel A) and vertebral artery blood flow (Δ%; panel B) in girls (white circles) and women (black squares); *within subject change from baseline, *P *<* *0.05.

ICA flow increased with hypoxia (*F*(1,17) = 9.645, *P *<* *0.01), but was consistently higher in the girls compared to women (*F*(1,17) = 10.104, *P *<* *0.01). When expressed as a percentage change the increase in ICA flow after 60 HYP was similar in girls and women (36% vs. 38%, respectively; see Fig. 3). Elevations in VA mean flow were also noted after 60 HYP (*F*(1,18) = 25.368, *P *<* *0.01), which did not differ between groups. In the girls, VA mean flow increased from 86 ± 21 mL·min^−1^ at baseline to 109 ± 37 mL·min^−1^ at 60 HYP. In women, VA mean flow increased from 81 ± 41 mL·min^−1^ at baseline to 112 ± 50 mL·min^−1^ at 60 HYP.

**Figure 4:**
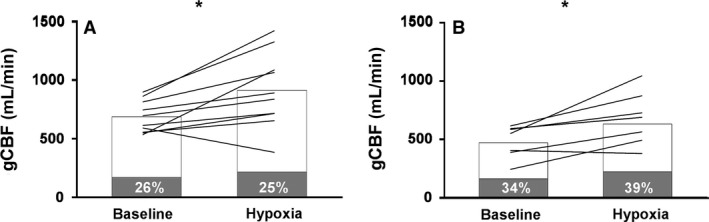
Global cerebral blood flow distribution at baseline and after 60 min of hypoxia in girls (A) and women (B). The gray bars represent vertebral artery contribution and the white bars represent internal carotid artery contribution; *within subject change from baseline, *P *<* *0.05.

Global CBF increased with hypoxia (*F*(1,17) = 16.969, *P *<* *0.001), in a similar manner in girls and women. When the contribution of extracranial artery flow to gCBF was considered, this was accounted for by ICA flow contributing to 75% of gCBF in the girls, but just 61% in the women at 60 HYP, while VA flow contributed 25% of gCBF in the girls, but 39% in the women (see Fig. 4). The increase in gCBF was not correlated with the decline in SpO_2_ (*r* = −0.101, *P *=* *0.680) or P_ET_CO_2_ (*r* = 0.458, *P *=* *0.064).

### Cerebral blood velocity and arterial diameter

ICA blood velocity did not change in response to 60 HYP, whilst there was a significant increase in VA blood velocity (*F*(1,18) = 6.252, *P *<* *0.05) which was similar between girls and women (see Table 3). In women, VA blood velocity significantly increased from baseline following 60 HYP (*P *<* *0.05), though in girls this failed to reach significance. There was a significant main effect for ICA diameter (*F*(1,17) = 8.237, *P *<* *0.05) and interaction (*F*(1,17) = 6.824, *P *<* *0.05), but not age. In women, ICA diameter significantly increased from baseline following 60 HYP (*P *<* *0.05), though in girls this failed to reach significance (see Table 3). VA diameter increased with hypoxia (*F*(1,18) = 40.477, *P *<* *0.05) similarly between girls and women (see Table 3).

**Table 3:** Internal carotid and vertebral artery diameter at baseline (BL) and following 60 min (60 HYP) of hypoxic exposure in girls and women. Data are mean (±SD)

		BL	60 HYP
ICA diameter (cm)	Girls (*n *=* *10)	0.471 (0.066)	0.506 (0.049)
	Women (*n *=* *9)	0.419 (0.045)	0.447 (0.044)^a^
VA diameter (cm)	Girls (*n *=* *10)	0.374 (0.040)	0.410 (0.055)^a^
	Women (*n *=* *10)	0.384 (0.080)	0.422 (0.080)^a^
ICA velocity (cm·sec^−1^)	Girls (*n *=* *10)	48.8 (10.8)	56.8 (20.9)
	Women (*n *=* *9)	37.4 (11.0)	41.4 (14.4)
VA velocity (cm·sec^−1^)	Girls (*n *=* *10)	25.6 (3.7)	26.6 (4.6)
	Women (*n *=* *10)	21.8 (6.1)	25.4 (7.2)^a^

a within subject change from baseline, *P *<* *0.05.

## Discussion

We report, for the first time in pre‐ to early‐pubertal girls, that cerebral perfusion of the extracranial arteries increases in response to acute normobaric hypoxia. Interestingly, the distribution of gCBF favors ICA flow in the girls, but VA flow in women. Additionally, increases in ${\dot{V}}_{E}$, *V*_T_/*T*_I_ and decreases in SpO_2_ and P_ET_CO_2_ are comparable between girls and women, although the pattern of breathing differed, with an increased *V*_T_ in girls and an increased f_R_ in women. These findings suggest that developmental differences exist in the way increased gCBF and ventilatory parameters are mediated in response to acute hypoxia.

### Ventilatory responses to hypoxia in children

Similar to the findings of Kohler and colleagues (Kohler et al. 2008) in hypobaric hypoxia, we also observe comparable ${\dot{V}}_{E}$ and *V*_T_/*T*_I_ responses between girls and women. Although we did not directly assess hypoxic chemosensitivity, our findings provide support that hypoxic ventilatory chemoreflex response is not age dependent. ${\dot{V}}_{E}$ following 5 min of hypoxia in girls was elevated by an appreciable 25%, though this increase was nonsignificant. This is likely explained by the large inter‐individual variation observed at this time point, since one girl had a much greater increase in ${\dot{V}}_{E}$. Interestingly, the decline in P_ET_CO_2_ was no more than for other girls, who did not show such a marked increase in ${\dot{V}}_{E}$; most likely because the increase in ${\dot{V}}_{E}$ was a result of increased *V*_T_ as opposed to a more elevated f_R_. Children are notoriously ‘noisy’ breathers, so observing high interchild variability is not unusual (Potter et al. 1999). Our baseline P_ET_CO_2_ data is within normal resting range (Cooper et al. 1987) and although children regulate PaCO_2_ at a lower set point and breathe with a higher ${\dot{V}}_{E}$/$\dot{V}O_{2}$ at rest and during exercise (McMurray et al. 2003), we found no child‐adult difference in P_ET_CO_2_ following acute hypoxia. Additionally, ${\dot{V}}_{E}$/$\dot{V}O_{2}$ remained constant in the girls, but increased in women following 5 and 30 min of hypoxia. Theoretically, if hypoxia‐induced hyperventilation in the child induced further declines in P_ET_CO_2_, this may result in hypocapnic‐induced cerebral vasoconstriction and consequent attenuation of CBF (Kety and Schmidt 1946). Yet, we do not observe this; therefore, since the variability of the chemoreflex changes in arterial blood gases can influence CBF (Willie et al. 2014; Hoiland et al. 2016), the comparable changes in blood gases might explain the similar changes in CBF and hence presumably adequate O_2_ delivery to the brain. Likewise, although directionally similar, the variability within the chemoreflex responses likely underpin the variability in the CBF responses.

### Cerebral hemodynamic responses to hypoxia

Cerebral perfusion is known to be developmentally mediated, and peaks between the ages of 5–10 years (Leung et al. 2016). Consequently, these elevations in resting CBF have been linked to a reduced cerebrovascular reserve in response to hypercapnia (Leung et al. 2016), though little is known about the response to hypoxia. Under hypobaric hypoxic conditions, an increase in anterior CBF has been observed (indexed by middle and anterior cerebral blood velocity; Gavlak et al. 2013). Likewise, we report significantly elevated anterior flow of the extracranial vessels (indexed by the ICA); however, we also report significant elevations in extracranial posterior flow (indexed by the VA) which was not observed in the intracranial vessels (indexed by basilar and posterior cerebral blood velocity; Gavlak et al. 2013). It is possible that the use of transcranial Doppler cerebral velocity has clouded our understanding because of the inability to account for diameter changes. Similar to responses to hypoxia in adults (Wilson et al. 2011), we report significantly increased VA diameter, and although not significant, a trend toward an increase in mean ICA diameter in girls. Thus, despite a potentially lower cerebrovascular reserve, girls still demonstrate the ability to significantly increase both anterior and posterior flow under hypoxic conditions. This response is likely paramount for increasing cerebral O_2_ delivery, protecting the young brain against otherwise detrimental effects of hypoxia.

In adults, acute poikilocapnic hypoxia causes elevations in both ICA and VA blood flow, though the percentage increase in VA flow (~37%) is much higher than in the ICA (~18%) (Lewis et al. 2014). It is believed that this is likely because the cardiovascular and respiratory control centers located in the brainstem require greater oxygenation and hence an increase in posterior cerebral flow (Ogoh et al. 2013; Lewis et al. 2014). Instead, we report a preferential dependence of increased ICA flow in girls, supporting the observations described by Gavlak et al. (2013), using transcranial Doppler. It has been suggested that a smaller rise in posterior cerebral flow may compromise oxygen delivery to the parieto‐occiptal cerebellar brain tissue, which may correspond to symptoms of nausea and dizziness (Gavlak et al. 2013). This may explain why children typically report a higher prevalence of AMS (Kohler et al. 2008), although no difference in AMS was noted between the girls and women in this study, likely due to its short‐term nature. Although speculative, it might be that the local metabolic demand (due to development) is less in the brainstem regions in children and hence could explain why the relative increase in VA flow is less when compared to adults. Although under normoxic resting conditions, the distribution of anterior and posterior blood velocity in the intracranial vessels has been shown to be similar between children and adults (Schöning et al. 1993). Since both resting diameter and the relative dilation of the VA was similar between children and adults, it would seem these differences are not explained by anatomical influences on vessel structure.

During conditions of isocapnic hypoxia, an appreciable increase in CBF does not occur until PaO_2_ is reduced below 50 mmHg (SpO_2_ <80%; reviewed in (Hoiland et al. 2016; Willie et al. 2014). What is interesting in the current study is that CBF was still markedly elevated when SpO_2_ was ~80%, even with evident hypocapnia (P_ET_CO_2_ ~26 mmHg). Normally this level of hypocapnia alone would be expected to *reduce* CBF by ~30–40% (assuming a normal hypocapnic reactivity of 2–4% reduction in CBF per mmHg reduction in P_ET_CO_2_). It is possible that it is reductions in oxygen content and/or hemoglobin deoxygenation (rather than arterial blood gases per se) during poikilocapnic hypoxia that are mediating elevations in CBF as a way of maintaining oxygen delivery. This topic, and related mechanisms of action, has been recently reviewed in detail elsewhere (Hoiland et al. 2016).

### Limitations

We chose to assess hypoxia under normobaric pressure in the interest of participant safety. As such, our findings cannot be directly related to environmental (hypobaric) hypoxia. The physiological effects of normobaric versus hypobaric hypoxia has been a topic of recent debate. SpO_2_ and ${\dot{V}}_{E}$ appear to be lower following short‐term exposure to hypobaric compared to normobaric hypoxia (Coppel et al. 2015), which may have led to an underestimation of ${\dot{V}}_{E}$ and an overestimation of SpO_2_ in our study if these results were extrapolated to environmental hypoxia. Similarly, these findings are limited to an acute bout (1 h) of hypoxia. Nothing is known of the extracranial blood flow response to longer‐term exposure and further investigation is needed to extend our understanding of hypoxic exposure in the child. Menstrual cycle phase in women was not controlled for. Varying hormonal stages of the menstrual cycle are known to influence ${\dot{V}}_{E}$ and *V*_T_/*T*_I_ (Schoene et al. 1981), thus, if we had standardized for menstrual cycle phase, our results may have varied slightly.

In conclusion, this investigation provides a novel insight into the extracranial blood flow and diameter, and ventilatory responses to an acute 1 h bout of normobaric hypoxia in children. Significant increases in ICA, VA, and gCBF were noted in girls, and VA and gCBF in women, with regional distribution of CBF favoring the anterior circulation in girls and the posterior circulation in women. Furthermore, increases in ${\dot{V}}_{E}$ and *V*_T_/*T*_I_, and declines in SpO_2_ and P_ET_CO_2_ were comparable between girls and women, though the increase in ${\dot{V}}_{E}$ was mediated via an increase in *V*_T_ in girls, and an increased f_R_ in women. These results imply that the fundamentals of increasing CBF and ventilatory parameters on exposure to hypoxia are similar between ages, but the way in which these responses are mediated are developmentally divergent.

## Conflict of Interest

The authors disclose no perceived or potential conflicts of interest.

## References

[phy213372-bib-0001] Ainslie, P. N. , and R. L. Hoiland . 2014 Transcranial Doppler ultrasound: valid, invalid, or both? J. Appl. Physiol. 117:1081–1083. 25257879 10.1152/japplphysiol.00854.2014

[phy213372-bib-0002] Cooper, D. M. , M. R. Kaplan , L. Baumgarten , D. Weiler‐Ravell , B. J. Whipp , and K. Wasserman . 1987 Coupling of ventilation and CO2 production during exercise in children. Pediatr. Res. 21:568–572. 3110725 10.1203/00006450-198706000-00012

[phy213372-bib-0003] Coppel, J. , P. Hennis , E. Gilbert‐Kawai , and M. P. Grocott . 2015 The physiological effects of hypobaric hypoxia versus normobaric hypoxia: a systematic review of crossover trials. Extrem. Physiol. Med. 4:2. 25722851 10.1186/s13728-014-0021-6 PMC4342204

[phy213372-bib-0004] Gaultier, C. L. , L. Perret , M. Boule , A. Buvry , and F. Girard . 1981 Occlusion pressure and breathing pattern in healthy children. Respir. Physiol. 46:71–80. 7330494 10.1016/0034-5687(81)90069-4

[phy213372-bib-0005] Gavlak, J. C. , J. Stocks , A. Laverty , E. Fettes , R. Bucks , S. Sonnappa , et al. 2013 The Young Everest Study: preliminary report of changes in sleep and cerebral blood flow velocity during slow ascent to altitude in unacclimatised children. Arch. Dis. Child. 98:356–362. 23471157 10.1136/archdischild-2012-302512 PMC3625826

[phy213372-bib-0006] Gratas‐Delamarche, A. , J. Mercier , M. Ramonatxo , J. Dassonville , and C. Prefaut . 1993 Ventilatory response of prepubertal boys and adults to carbon dioxide at rest and during exercise. Eur. J. Appl. Physiol. 66:25–30. 10.1007/BF00863395 8425509

[phy213372-bib-0007] Hoiland, R. L. , and P. H. Ainslie . 2016 CrossTalk proposal: the middle cerebral artery diameter does change during alterations in arterial blood gases and blood pressure. J. Physiol. 594:4073–4075. 27010010 10.1113/JP271981 PMC4806217

[phy213372-bib-0008] Hoiland, R. L. , A. R. Bain , M. G. Rieger , D. M. Bailey , and P. N. Ainslie . 2016 Hypoxemia, oxygen content, and the regulation of cerebral blood flow. Am. J. Physiol. Regul. Integr. Comp. Physiol. 310:R398–R413. 26676248 10.1152/ajpregu.00270.2015 PMC4796739

[phy213372-bib-0009] Hoiland, R. L. , A. R. Bain , M. M. Tymko , M. G. Rieger , C. A. Howe , C. K. Willie , et al. 2017 Adenosine receptor dependent signaling is not obligatory for normobaric and hypobaric hypoxia‐induced cerebral vasodilation in humans. J. Appl. Physiol.. https://doi.org/10.1152/japplphysiol.00840.2016. 10.1152/japplphysiol.00840.2016 PMC5407198 28082335

[phy213372-bib-0010] Honda, Y. , Y. Ohyabu , H. Masuyama , Y. Nishibayashi , R. Maruyama , Y. Tanaka , et al. 1986 Hypercapnic and hypoxic ventilatory responses during growth. Jpn. J. Physiol. 36:177–187. 3088307 10.2170/jjphysiol.36.177

[phy213372-bib-0011] Imray, C. , C. Chan , A. Stubbings , H. Rhodes , S. Patey , M. H. Wilson , et al. 2014 Time course variations in the mechanisms by which cerebral oxygen delivery is maintained on exposure to hypoxia/altitude. High Alt. Med. Biol. 15:21–27. 24559404 10.1089/ham.2013.1079

[phy213372-bib-0012] Kety, S. S. , and C. F. Schmidt . 1946 The effects of active and passive hyperventilation on cerebral blood flow, cerebral oxygen consumption, cardiac output, and blood pressure of normal young men. J. Clin. Invest. 25:107–119. 21016304

[phy213372-bib-0013] Kohler, M. , S. Kriemler , E. M. Wilhelm , H. Brunner‐LaRocca , M. Zehnder , and K. E. Bloch . 2008 Children at high altitude have less nocturnal periodic breathing than adults. Eur. Respir. J. 32:189–197. 18287125 10.1183/09031936.00119807

[phy213372-bib-0014] Kriemler, S. , T. Radtke , F. Bürgi , J. Lambrecht , M. Zehnder , and H. P. Brunner‐La Rocca . 2015 Short‐term cardiorespiratory adaptation to high altitude in children compared with adults. Scand. J. Med. Sci. Sports 26:147–155. 25648726 10.1111/sms.12422

[phy213372-bib-0015] Leung, J. , P. D. Kosinski , P. L. Croal , and A. Kassner . 2016 Developmental trajectories of cerebrovascular reactivity in healthy children and young adults assessed with magnetic resonance imaging. J. Physiol. 594:2681–2689. 26847953 10.1113/JP271056 PMC4865568

[phy213372-bib-0016] Lewis, N. C. , L. Messinger , B. Monteleone , and P. N. Ainslie . 2014 Effect of acute hypoxia on regional cerebral blood flow: effect of sympathetic nerve activity. J. Appl. Physiol. 116:1189–1196. 24610534 10.1152/japplphysiol.00114.2014 PMC4098059

[phy213372-bib-0017] Marcus, C. L. , W. B. Glomb , D. J. Basinski , S. L. Davisdon , and T. G. Keens . 1994 Developmental pattern of hypercapnic and hypoxic ventilatory responses from childhood to adulthood. J. Appl. Physiol. 76:314–320. 8175523 10.1152/jappl.1994.76.1.314

[phy213372-bib-0018] McMurray, R. G. , C. Bagget , M. Pennell , S. Bangdiwala , and J. Harrell . 2003 Gender differences in ventilatory responses of youth are related to exercise intensity. Port J. Sport Sci. 3:101–102.

[phy213372-bib-0019] Ogoh, S. , K. Sato , H. Nakahara , K. Okazaki , A. W. Subudhi , and T. Miyamoto . 2013 Effect of acute hypoxia on blood flow in vertebral and internal carotid arteries. Exp. Physiol. 98:692–698. 23143991 10.1113/expphysiol.2012.068015

[phy213372-bib-0020] Potter, C. R. , D. J. Childs , W. Houghton , and N. Armstrong . 1999 Breath‐to‐breath, “noise” in the ventilatory and gas exchange responses of children to exercise. Eur. J. Appl. Physiol. 80:118–124. 10.1007/s004210050567 10408322

[phy213372-bib-0021] Rasmussen, A. R. , C. Wohlfahrt‐Veje , K. T. de Renzy‐Martin , C. P. Hagen , J. Tinggaard , A. Mouritsen , et al. 2015 Validity of self‐assessment of pubertal maturation. Pediatrics 135:86–93. 25535262 10.1542/peds.2014-0793

[phy213372-bib-0022] Roach, R. C. , P. Bartsch , O. Oelz , and P. H. Hackett . 1993 The Lake Louise acute mountain sickness scoring system Pp. 272–274 in Sutton J. R., Houston C. S. and Coates G., eds. Hypoxia and Molecular Medicine. Queen City Press, Burlington, VT.

[phy213372-bib-0023] Sampson, J. B. , A. Cymerman , R. L. Burse , J. T. Maher , and P. B. Rock . 1983 Procedures for the measurement of acute mountain sickness. Aviat. Space Environ. Med. 54:1063–1073. 6661120

[phy213372-bib-0024] Schoene, R. B. , H. T. Robertson , D. J. Pierson , and A. P. Peterson . 1981 Respiratory drives and exercise in menstrual cycles of athletic and nonathletic women. J. Appl. Physiol. 50:1300–1305. 7263392 10.1152/jappl.1981.50.6.1300

[phy213372-bib-0025] Schöning, M. , and B. Hartig . 1996 Age dependence of total cerebral blood flow volume from childhood to adulthood. J. Cerebr. Blood F. Met. 16:827–833. 10.1097/00004647-199609000-00007 8784227

[phy213372-bib-0026] Schöning, M. , M. Staab , J. Walter , and G. Niemann . 1993 Transcranial color duplex sonography in childhood and adolescence. Age dependence of flow velocities and waveform parameters. Stroke 24:1305–1309. 8362422 10.1161/01.str.24.9.1305

[phy213372-bib-0027] Thomas, K. N. , N. C. Lewis , B. G. Hill , and P. N. Ainslie . 2015 Technical recommendations for the use of carotid duplex ultrasound for the assessment of extracranial blood flow. Am J Physiol‐Reg I 309: R707–R720. 10.1152/ajpregu.00211.2015 26157060

[phy213372-bib-0028] Tontisirin, N. , S. L. Muangman , P. Suz , C. Pihoker , D. Fisk , A. Moore , et al. 2007 Early childhood gender differences in anterior and posterior cerebral blood flow velocity and autoregulation. Pediatrics 119:e610–e615. 17283178 10.1542/peds.2006-2110

[phy213372-bib-0029] Weil, J. V. , E. Byrne‐Quinn , I. E. Sodal , W. O. Friesen , B. Underhill , G. F. Filley , et al. 1970 Hypoxic ventilatory drive in normal man. J. Clin. Invest. 49:1061. 5422012 10.1172/JCI106322 PMC322574

[phy213372-bib-0030] Willie, C. K. , D. B. Macleod , A. D. Shaw , K. J. Smith , Y. C. Tzeng , N. D. Eves , et al. 2012 Regional brain blood flow in man during acute changes in arterial blood gases. J. Physiol. 590:3261–3275. 22495584 10.1113/jphysiol.2012.228551 PMC3459041

[phy213372-bib-0031] Willie, C. K. , Y. C. Tzeng , J. A. Fisher , and P. N. Ainslie . 2014 Integrative regulation of human brain blood flow. J. Physiol. 592:841–859. 24396059 10.1113/jphysiol.2013.268953 PMC3948549

[phy213372-bib-0032] Wilson, M. H. , M. E. Edsell , I. Davagnanam , S. P. Hirani , D. S. Martin , D. Z. Levett , et al. 2011 Cerebral artery dilation maintains cerebral oxygenation at extreme altitude and in acute hypoxia – an ultrasound and MRI study. J. Cerebr. Blood F. Met. 31:2019–2029. 10.1038/jcbfm.2011.81 PMC3208157 21654697

[phy213372-bib-0033] Wolff, H. G. , W. G. Lennox , and M. B. Allen . 1930 Cerebral circulation: XII. The effect on pial vessels of variations in the oxygen and carbon dioxide content of the blood. Arch. Neuro. Psychiatr. 23:1097–1120.

